# Patient Anxiety in Endoscopy: A Comparative Analysis of Single vs. Dual Procedure Effects

**DOI:** 10.7759/cureus.57237

**Published:** 2024-03-30

**Authors:** Tuna Albayrak, Ayşegül Torun Göktaş, Selin Eyüpoğlu, Ali Muhtaroğlu, Ahmet Cumhur Dulger

**Affiliations:** 1 Department of Anesthesiology and Reanimation, Giresun University Faculty of Medicine, Giresun, TUR; 2 Department of Anesthesiology and Reanimation, Giresun İlhan Özdemir State Hospital, Giresun, TUR; 3 Department of Anesthesiology and Critical Care, Giresun Training and Research Hospital, Giresun, TUR; 4 Department of General Surgery, Giresun University Faculty of Medicine, Giresun, TUR; 5 Department of Gastroenterology, Giresun University Faculty of Medicine, Giresun, TUR

**Keywords:** beck anxiety inventory scale, preoperative anxiety, patient anxiety, colonoscopy, gastroscopy

## Abstract

Aim: This study aimed to investigate the difference in anxiety levels between patients undergoing gastroscopy only and those subjected to both gastroscopy and colonoscopy. Despite known preoperative anxiety impacts, no prior research has compared these specific patient groups.

Materials and methods: A total of 150 patients were divided equally into two groups: Group I, undergoing gastroscopy only, and Group II, undergoing gastroscopy and colonoscopy. Inclusion criteria were patients in the age range 18-70 years and having an ASA (American Society of Anesthesiologists) physical status classification of I-III. Exclusion criteria were patients outside the age range, and patients with hearing disorders, psychiatric disorders, dementia, or recent anxiolytic drug use. Anxiety was analysed using the Beck Anxiety Inventory Scale before procedures, without any premedication.

Results: Patients in Group II had significantly higher anxiety levels, with particular increases noted in symptoms such as leg weakness and tremors, inability to relax, and fears of adverse events and death. These results highlighted a considerable elevation in anxiety among patients anticipating or undergoing combined endoscopic procedures.

Discussion: The findings revealed that undergoing combined gastroscopy and colonoscopy procedures significantly elevated patient anxiety levels compared to gastroscopy alone. This suggests a critical need for healthcare providers to implement more strong preoperative counselling and anxiety reduction strategies for patients facing multiple procedures. Addressing this increased anxiety could lead to better patient experiences, reduced procedural complications, and improved satisfaction and outcomes.

## Introduction

Endoscopic procedures, especially gastroscopy and colonoscopy, are cornerstone diagnostic tools in gastroenterology, offering invaluable insights into gastrointestinal tract health [1,2]. While these procedures are widely considered safe and effective, the anticipation of undergoing endoscopy can provoke significant anxiety in patients [3-5]. Anxiety related to medical procedures is well-documented, influencing not only the patient's psychological well-being but also potentially impacting procedural outcomes and recovery processes. This emphasises the importance of understanding and addressing patient anxiety within the context of endoscopic examinations [6-9].

Recent literature highlights the multifaceted nature of preoperative anxiety, identifying factors such as fear of diagnosis, procedural pain, and the general unknown as critical contributors to increased patient distress. The Beck Anxiety Inventory (BAI), a reliable self-reporting tool, has been extensively used to quantify levels of anxiety, offering clinicians a practical means to assess and address this critical aspect of patient care [10,11].

Despite the recognised impact of anxiety on patient outcomes, there exists a gap in the literature regarding the comparative analysis of anxiety levels between patients undergoing single versus combined endoscopic procedures. This oversight is remarkable, given the increasing prevalence of patients recommended for gastroscopy and colonoscopy during a single clinical visit. Such an approach, while efficient, raises questions about its psychological impact on patients. Are individuals more anxious about undergoing two procedures simultaneously, and if so, what are the implications for clinical practice?

The study results in this field touch upon two main points. Firstly, they offer a deeper understanding of patient experiences and anxieties related to endoscopic procedures, potentially revealing a need for enhanced communication, preparation, and support strategies. Secondly, they provide a foundation for developing more tailored anxiety management interventions, which could improve patient compliance, satisfaction, and overall procedural outcomes.

In light of these thoughts, our study aimed to fill the existing research gap by comparing anxiety levels in patients undergoing gastroscopy alone to those subjected to both gastroscopy and colonoscopy. Through this investigation, we sought to contribute valuable insights into the psychological impacts of endoscopic procedures, with the ultimate goal of improving patient care and procedural efficacy in gastroenterology.

## Materials and methods

The local ethics committee of Giresun Training and Research Hospital, Giresun, Turkey, approved the study protocol. A patient information form was provided and the BAI Scale was applied to each patient before being brought to the endoscopy room. This study was conducted on the relevant ethical principles of the Declaration of Helsinki, revised in 2013. The study was conducted at Giresun Training and Research Hospital in Giresun province in Turkey.

This prospective study was conducted on 150 patients who underwent upper and lower gastrointestinal endoscopy and presented with dyspeptic complaints to our institution's general surgery, internal medicine, and gastroenterology outpatient clinics between July 2023 and December 2023. These patients were divided into two groups: 75 underwent upper gastrointestinal endoscopy only, and 75 underwent both upper and lower gastrointestinal endoscopy in the same session. The combination of midazolam and propofol was preferred for sedation before the procedure. Following a bolus dose of 0.05 mg/kg midazolam, 0.05 mg/kg/h infusion was started and titrated when necessary. Following a 1 mg/kg bolus dose of propofol, a 2.5 mg/kg/h infusion was started and dose titration was provided when necessary [12]. Patients' demographic information, such as age and gender, was obtained from patient records. Before the procedures, the BAI Scale was applied to the patients to measure their anxiety.

The BAI includes 21 questions, each answer being scored on a scale value of 0 (not at all) to 3 (severely). Higher total scores represent more severe anxiety symptoms. The standardised cutoffs are 0-7: minimal, 8-15: mild, 16-25: moderate, and 26-63: severe. Patients' demographic information, such as age and gender, was obtained from patient records. Among the answers given in the BAI Scale, “minimal” and “mild” were considered together, while “moderate” and “severe” were also evaluated together statistically. The BAI scale is shown in detail in Table 1.

**Table 1:** Beck Anxiety Inventory [13]

	Not at all	Mildly, but it didn’t bother me much	Moderately – it wasn’t pleasant at times	Severely – it bothered me a lot
Numbness or tingling	0	1	2	3
Feeling hot	0	1	2	3
Wobbliness in legs	0	1	2	3
Unable to relax	0	1	2	3
Fear of the worst happening	0	1	2	3
Dizzy or lightheaded	0	1	2	3
Heart pounding/racing	0	1	2	3
Unsteady	0	1	2	3
Terrified or afraid	0	1	2	3
Nervous	0	1	2	3
Feeling of choking	0	1	2	3
Hands trembling	0	1	2	3
Shaky/unsteady	0	1	2	3
Fear of losing control	0	1	2	3
Difficulty in breathing	0	1	2	3
Fear of dying	0	1	2	3
Scared	0	1	2	3
Indigestion	0	1	2	3
Faint/lightheaded	0	1	2	3
Face flushed	0	1	2	3
Hot/cold sweats	0	1	2	3
Column sum				

Statistical analysis

Statistical analyses were performed with IBM SPSS Statistics for Windows, Version 23, (Released 2015; IBM Corp., Armonk, New York, United States). Normality distributions of quantitative data were made with the Kolmogorov-Smirnov test. A comparison of data that did not comply with normal distribution was made with the Mann-Whitney U test. A Pearson's chi-square test and the Bonferroni correction were used to compare qualitative data. The relationship between the data was made with Pearson's correlation test. Data are presented as median (minimum-maximum) and n (%). The statistical significance value was accepted as p<0.05.

## Results

One hundred fifty patients were included in the study. The patients were divided into two groups: 75 in group 1, where upper gastrointestinal endoscopy was performed alone, and 75 in group 2, where upper and lower gastrointestinal endoscopy were performed together. A total of 56.66% (85 patients) were female and 43.34% (65 patients) were male. Of the patients who underwent upper gastrointestinal endoscopy only, 58.66% (44) were female, and 41.34% (31) were male. Of the patients who underwent upper and lower gastrointestinal endoscopy, 54.66% (41) were female, and 45.34% (34) were male. Demographic data of the patients are shown in Table 2.

**Table 2:** Comparison of characteristics of patients between the two groups Descriptive statistics are shown as n (%). The * symbol represents Pearson's Chi-Square analysis.

Characteristics of patients	Group 1	Group 2	P*
Gender			
Female	44 (58.66%)	41 (54.66%)	0.621
Male	31 (41.34%)	34 (45.34%)	
Marital status			
Married	54 (70%)	68 (90%)	0.003
Single	21 (30%)	7 (10%)	
Educational status			
Primary education	36 (48%)	48 (64%)	0.134
High school	17 (22.7%)	13 (17.3%)	
University	22 (29.3%)	14 (18.7%)	
Working status			
Working	29 (38.7%)	24 (32%)	0.393
Not working	46 (61.3%)	51 (68%)	
Economical situation			
Worse	1 (1,3%)	3 (4%)	0.141
Middle	42 (56%)	51 (68%)	
Good	30 (40%)	21 (28%)	
Very good	2 (2.7%)	0 (0%)	
Have you had an endoscopy before?			
No	40 (53.3%)	31 (41.3%)	0.141
Yes	35 (46.7%)	44 (58.7%)	

The information provided to the patients, their perceptions of the timing of this information, and their anxiety levels measured by the Beck score were examined (Table 3).

**Table 3:** Impact of procedural information on patient perception and anxiety levels Descriptive statistics are shown as n (%). The * symbol represents Pearson's Chi-Square analysis.

Procedural information	Group 1	Group 2	P*
Was written or verbal information given about the procedure?			
No	6 (8)	0 (0)	0.012
Yes	69 (92)	75 (100)	
Do you think this was a reasonable period?			
No	27 (36)	34 (45.3)	0.245
Yes	48 (64)	41 (54.7)	
Beck Score Group			
Mild	68 (90.7)	54 (72)	0.013
Moderate	5 (6.7)	13 (17.3)	
Severe	2 (2.7)	8 (10.7)	

The answers given to the questions regarding wobbliness in legs, inability to relax, fear of the worst happening, dizziness or lightheadedness, unsteadiness, nervousness, a feeling of dying, being scared, facial flushing, and hot/cold sweats on the Beck scale were significantly in favour of moderate/severe in the second group (Table 4).

**Table 4:** Responses to questions on the Beck Anxiety Inventory Scale Descriptive statistics are shown as n (%). The * symbol represents Pearson's Chi-Square analysis.

Questions and responses	Group 1	Group 2	P*
Numbness or tingling			
None/Mild	69 (92)	63 (84)	0.132
Moderate/Severe	6 (8)	12 (16)	
Feeling hot			
None/Mild	69 (92)	61 (81.3)	0.055
Moderate/Severe	6 (8)	14 (18.7)	
Wobbliness in legs			
None/Mild	69 (92)	55 (73.3)	0.003
Moderate/Severe	6 (8)	20 (26.7)	
Unable to relax			
None/Mild	71 (94.7)	61 (81.3)	0.012
Moderate/Severe	4 (5.3)	14 (18.7)	
Fear of the worst happening			
None/Mild	70 (93.3)	62 (82.7)	0.044
Moderate/Severe	5 (6.7)	13 (17.3)	
Dizzy or lightheaded			
None/Mild	69 (92)	59 (78.7)	0.021
Moderate/Severe	6 (8)	16 (21.3)	
Heart pounding/racing			
None/Mild	69 (92)	63 (84)	0.132
Moderate/Severe	6 (8)	12 (16)	
Unsteady			
None/Mild	74 (98.7)	67 (89.3)	0.016
Moderate/Severe	1 (1.3)	8 (10.7)	
Terrified or afraid			
None/Mild	72 (96)	66 (88)	0.071
Moderate/Severe	3 (4)	9 (12)	
Nervous			
None/Mild	61 (81.3)	42 (56)	0.001
Moderate/Severe	14 (18.7)	33 (44)	
Feeling of choking			
None/Mild	67 (89.3)	67 (89.3)	1.000
Moderate/Severe	8 (10.7)	8 (10.7)	
Hands trembling			
None/Mild	71 (94.7)	70 (93.3)	0.731
Moderate/Severe	4 (5.3)	5 (6.7)	
Shaky/unsteady			
None/Mild	73 (97.3)	70 (93.3)	0.246
Moderate/Severe	2 (2.7)	5 (6.7)	
Fear of losing control			
None/Mild	74 (98.7)	69 (92)	0.053
Moderate/Severe	1 (1.3)	6 (8)	
Difficulty in breathing			
None/Mild	72 (96)	66 (88)	0.071
Moderate/Severe	3 (4)	9 (12)	
Feeling of dying			
None/Mild	74 (98.7)	64 (85.3)	0.003
Moderate/Severe	1 (1.3)	11 (14.7)	
Scared			
None/Mild	73 (97.3)	64 (85.3)	0.009
Moderate/Severe	2 (2.7)	11 (14.7)	
Indigestion			
None/Mild	47 (62.7)	43 (57.3)	0.505
Moderate/Severe	28 (37.3)	32 (42.7)	
Faint/lightheaded			
None/Mild	74 (98.7)	71 (94.7)	0.172
Moderate/Severe	1 (1.3)	4 (5.3)	
Face flushed			
None/Mild	75 (100)	64 (85.3)	0.001
Moderate/Severe	0 (0)	11 (14.7)	
Hot/cold sweats			
None/Mild	72 (96)	60 (80)	0.003
Moderate/Severe	3 (4)	15 (20)	

## Discussion

The findings of our study underscore a significant elevation in anxiety levels among patients undergoing combined gastroscopy and colonoscopy procedures, as evidenced by markedly higher scores in the BAI. Mainly, symptoms such as wobbliness in legs, an inability to relax, fear of the worst happening, dizziness, unsteadiness, nervousness, feelings of dying, being scared, facial flushing, and experiencing hot or cold sweats were notably more pronounced in the second group. These results are critical, suggesting that the anticipation or the thought of undergoing dual procedures exacerbates patient anxiety to a moderate or severe degree.

This observed increase in anxiety, especially with symptoms that have a physical manifestation, such as wobbliness in the legs and hot or cold sweats, highlights the profound psychosomatic response that the prospect of endoscopic procedures can evoke. It is well-documented in the literature that preoperative anxiety can adversely affect not only the patient's experience of medical procedures but also their outcomes, including increased pain perception, prolonged recovery times, and, in some cases, complications during the procedure itself [14-17].

Benzodiazepines such as midazolam, which have shown efficacy in reducing preoperative anxiety in patients undergoing endoscopic procedures, are widely used for their anxiolytic, amnesic and sedative properties, making them suitable for premedication. In particular, midazolam is favoured for its rapid onset and short duration of action. Studies have demonstrated its efficacy in reducing anxiety before surgical and diagnostic procedures [18].

In addition, dexmedetomidine, an α2-adrenoceptor agonist, is gaining popularity for its sedative and anxiolytic effects without significant respiratory depression. It has been successfully used in preoperative settings to reduce anxiety and has shown benefits in both adults and children undergoing surgical procedures [19].

The significant anxiety responses identified in our study, particularly about specific fears such as dying or experiencing the worst possible outcomes, raise important considerations for clinical practice. This level of anxiety may reflect underlying concerns about the procedures' risks, potential diagnoses, or the discomfort associated with the procedures themselves. Therefore, it becomes imperative for healthcare providers to recognise these specific patient anxieties and address them through comprehensive preoperative counselling and tailored anxiety reduction strategies. Additionally, considering the significant role that anxiety plays in procedural and post-procedural outcomes, future research could explore the effectiveness of various anxiety-reduction techniques, such as mindfulness, biofeedback, or the use of virtual reality environments to simulate procedural experiences before the actual procedure [20].

Comparatively, our findings suggest a need for an enhanced focus on the psychological preparation of patients scheduled for dual endoscopic procedures. This could involve more detailed discussions about what to expect during and after the procedures, providing written materials to reinforce verbal information, and, potentially, introducing relaxation techniques or other anxiety-reducing interventions before the procedure.

Study limitations

This study has some limitations. First, it uses self-reported anxiety scores, which might not always capture how people truly feel. Also, we didn't consider factors like past medical experiences or support from friends and family, which can affect anxiety. Our group of 150 patients might not represent everyone who goes through these procedures, limiting how widely our findings apply. Lastly, we didn't check how patients felt after their procedures, so we don't know how long their anxiety lasted. These points are important to keep in mind when thinking about our results.

## Conclusions

In conclusion, our study reveals that patients undergoing combined gastroscopy and colonoscopy experience significantly higher levels of anxiety compared to those undergoing gastroscopy alone. These findings underscore the importance of healthcare providers implementing comprehensive preoperative counselling and tailored anxiety management strategies, particularly for patients facing multiple procedures. By addressing and mitigating preoperative anxiety, we can enhance patient experiences, reduce procedural complications, and improve overall satisfaction and outcomes. Future research should continue to explore effective interventions to support patients through their endoscopic procedures.
